# Regression models for predicting physical and EQD_2_ plan parameters of two methods of hybrid planning for stage III NSCLC

**DOI:** 10.1186/s13014-021-01848-9

**Published:** 2021-06-27

**Authors:** Hao Wang, Yongkang Zhou, Wutian Gan, Hua Chen, Ying Huang, Yanhua Duan, Aihui Feng, Yan Shao, Hengle Gu, Qing Kong, Zhiyong Xu

**Affiliations:** 1 Institute of Modern Physics, Fudan University, Shanghai, China; 2 Department of Radiation Oncology, Zhongshan Hospital, Shanghai, China; 3 School of Physics and Technology, University of Wuhan, Wuhan, China; 4 Department of Radiation Oncology, Shanghai Chest Hospital, Shanghai Jiaotong University, Shanghai, China

**Keywords:** Physical plan parameter, EQD2 plan parameter, Regression model, Hybrid planning, Stage III NSCLC

## Abstract

**Background/purpose:**

To establish regression models of physical and equivalent dose in 2 Gy per fraction (EQD_2_) plan parameters of two kinds of hybrid planning for stage III NSCLC.

**Methods:**

Two kinds of hybrid plans named conventional fraction radiotherapy & stereotactic body radiotherapy (C&S) and conventional fraction radiotherapy & simultaneous integrated boost (C&SIB) were retrospectively made for 20 patients with stage III NSCLC. Prescription dose of C&S plans was 2 Gy × 30f for planning target volume of lymph node (PTV_LN_) and 12.5 Gy × 4f for planning target volume of primary tumor (PTV_PT_), while prescription dose of C&SIB plans was 2 Gy × 26f for PTV_LN_ and sequential 2 Gy × 4f for PTV_LN_ combined with 12.5 Gy × 4f for PTV_PT_. Regression models of physical and EQD_2_ plan parameters were established based on anatomical geometry features for two kinds of hybrid plans. The features were mainly characterized by volume ratio, min distance and overlapping slices thickness of two structures. The possibilities of regression models of EQD_2_ plan parameters were verified by spearman’s correlation coefficients between physical and EQD_2_ plan parameters, and the influence on the consistence of fitting goodness between physical and EQD_2_ models was investigated by the correlations between physical and EQD_2_ plan parameters. Finally, physical and EQD_2_ models predictions were compared with plan parameters for two new patients.

**Results:**

Physical and EQD_2_ plan parameters of PTV_LN_ CI_60Gy_ have shown strong positive correlations with PTV_LN_ volume and min distance_(PT to LN)_, and strong negative correlations with PTV_PT_ volume for two kinds of hybrid plans. PTV_(PT+LN)_ CI_60Gy_ is not only correlated with above three geometry features, but also negatively correlated with overlapping slices thickness_(PT and LN)_. When neck lymph node metastasis was excluded from PTV_LN_ volume, physical and EQD_2_ total lung V_20_ showed a high linear correlation with corrected volume ratio_(LN to total lung)._ Meanwhile, physical total lung mean dose (MLD) had a high linear correlation with corrected volume ratio_(LN to total lung)_, while EQD_2_ total lung MLD was not only affected by corrected volume ratio_(LN to total lung)_ but also volume ratio_(PT to total lung)._ Heart D_5_, D_30_ and mean dose (MHD) would be more susceptible to overlapping structure_(heart and LN)_. Min distance_(PT to ESO)_ may be an important feature for predicting EQD_2_ esophageal max dose for hybrid plans. It’s feasible for regression models of EQD_2_ plan parameters, and the consistence of the fitting goodness of physical and EQD_2_ models had a positive correlation with spearman’s correlation coefficients between physical and EQD_2_ plan parameters. For total lung V_20_, ipsilateral lung V_20_, and ipsilateral lung MLD, the models could predict that C&SIB plans were higher than C&S plans for two new patients.

**Conclusion:**

The regression models of physical and EQD_2_ plan parameters were established with at least moderate fitting goodness in this work, and the models have a potential to predict physical and EQD_2_ plan parameters for two kinds of hybrid planning.

**Supplementary Information:**

The online version contains supplementary material available at 10.1186/s13014-021-01848-9.

## Background

Definitive concurrent chemoradiotherapy (CCRT) has been recommended as the standard treatment for unresectable or medically inoperable stage III non-small cell lung cancer (NSCLC) [1–3]. Primary tumor (PT) and lymph node metastasis (LN) are treated by conventional fraction radiotherapy (CFRT) for these patients.

Two studies did a detailed analysis about locoregional failure in stage III NSCLC treated by CFRT, and showed that PT recurrence occurred more often than LN recurrence [4, 5]. Therefore, there is a rationale for PT dose escalation. The results of the CHISEL trial [6] suggested that implement of stereotactic body radiotherapy (SBRT) to PT and CFRT to LN could improve control of PT and further improve control of the entire region with two distinct target volumes. SBRT is suitable for PT dose escalation as there is a certain distance between PT and LN which could allow for dose drop. Meanwhile, Tighter margin of planning target volume (PTV) and steeper dose drop around PT could reduce the risk of toxicity events while accurate dose delivery to PT could be implemented.

It has been reported that two main methods for hybrid planning with different dose regimes could be used to PT and LN. First method has now been tried in clinic that implement CFRT before [7–10] or after [11] SBRT boost for LN and PT (called C&S plan). Second method was proposed by Peulen et al. [12] that LN was treated by partial fractions of CFRT, and then simultaneous integrated boost (SIB) was implemented for PT and LN (called C&SIB plan).

Both methods of hybrid planning may be challenging, because the dose conformability is affected by the dose superposition of the two sub-plans. The closer distance between PT and LN results in more significant interaction effect, which makes equivalent dose in 2 Gy per fraction (EQD_2_) evaluation of the hybrid plan more complicated. It seems more meaningful to evaluate the hybrid plan when EQD_2_ corrected dose of SBRT/SIB plan is superposed with the dose of CFRT plan. In addition, anatomical geometry features may compromise the optimization objectives of two steps of hybrid planning, which greatly increases the difficulty of step-by-step optimization and evaluation. The features are mainly characterized by volume ratio of two contour structures, minimum distance between two structures, overlapping slices thickness of two spatially separated structures, and the volume ratio of overlapping structure by two structures to one of them.

Although these methods of hybrid planning may provide more opportunities to stage III NSCLC CCRT, it’s still not very clear which kind of hybrid planning has more dosimetric advantages, especially EQD_2_ dose advantages when the patients have different anatomical geometry features. Meanwhile, it would be challenging to compare EQD_2_ plan parameters of different kinds of hybrid plans in clinical practice. To say the least, it would be time-consuming to complete different kinds of hybrid plans for comparison. In this work, multiple regression models based on anatomical geometry features were proposed to predict plan parameters of two kinds of hybrid planning, which was a meaningful attempt to solve the above problems.

## Materials and methods

### Patient selection

**Fig. 1:**
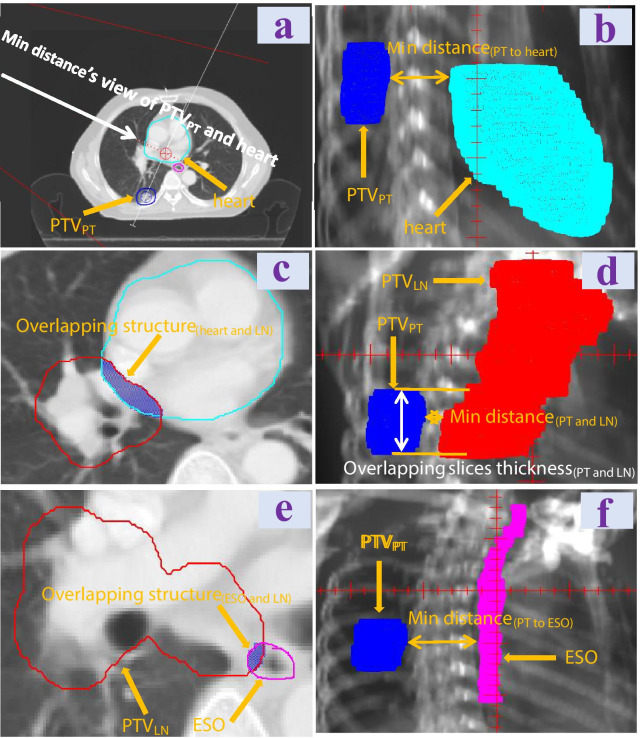
Anatomical geometry features shown on CT images and min distance view’s DRR images. **a** Min distance’s view of PTV_PT_ and heart (the view direction is vertical to the centers’ connection of PTV_PT_ and heart on CT slice); **b** Min distance_(PT to heart)_ shown on corresponding min distance view’s DRR image; **c** Volume ratio of overlapping structure_(heart and LN)_ to heart or PTV_LN_; **d** Min distance_(PT to LN)_ and overlapping slices thickness_(PT and LN)_ shown on corresponding min distance view’s DRR image; **e** Volume ratio of overlapping structure_(ESO and LN)_ to ESO; and **f** Min distance_(PT to ESO)_ shown on corresponding min distance view’s DRR image

A total of 20 patients with stage III NSCLC from January 2016 to June 2020 were retrospectively selected in our center, including 18 male patients and 2 female patients. Three patients received concurrent chemoradiation with CFRT for LN before SBRT boost for PT, as superior vena cava was oppressed by enlarged mediastinal lymph nodes, while the other seventeen patients received SBRT boost for PT before concurrent chemoradiation with CFRT for LN. Mean PTV_PT_ volume is 30.7 cc (10.9–100.1 cc), and mean PTV_LN_ volume is 199.5 cc (24.8–452.3 cc). Table 1 summarizes patients characteristics and anatomical geometry features. Anatomical geometry features were shown on CT and digitally reconstructured radiograph (DRR) images (Fig. 1).

**Table 1:** Patient characteristics

Factors		
Sex	Male	18
	Female	2
Age (years)	Median (range)	62 (46–71)
Location (lobe)	Right upper	10
	Right middle	1
	Right lower	5
	Left upper	3
	Left lower	1
Clinical stage	IIIA	8
	IIIB	12
Radiotherapy sequence	CFRT before SBRT boost	3
	CFRT after SBRT boost	17
PTV_PT_ volume (cc)	Median (range)	30.7 (10.9–100.1)
PTV_LN_ volume (cc)	Median (range)	199.5 (24.8–452.3)
Min distance_(PT to LN)_ (cm)	Median (range)	4.0 (1.2–7.1)
Overlapping slices thickness_(PT and LN)_ (cm)	Median (range)	1.9 (-4.5–6.1)
Volume ratio_(LN to total lung)_	Median (range)	0.07 (0.01–0.14)
Volume ratio_(LN to ipsilateral lung)_	Median (range)	0.12 (0.02–0.30)
Min distance_(PT to ESO)_ (cm)	Median (range)	5.83 (0.89–10.47)
Volume ratio of overlapping structure_(ESO and LN)_ to ESO	Median (range)	0.32 (0.00–0.87)
Min distance_(PT to heart)_ (cm)	Median (range)	4.27 (1.11–7.58)
Volume ratio of overlapping structure_(heart and LN)_ to heart	Median (range)	0.02 (0.00–0.05)
Volume ratio of overlapping structure_(heart and LN)_ to PTV_LN_	Median (range)	0.07 (0.01–0.24)

To make the presentation more concise, PT represented PTV_PT_, LN represented PTV_LN_, ESO represented esophagus, CFRT represented conventional fraction radiotherapy, and SBRT represented stereotactic body radiotherapy. The explanation of each anatomical geometric parameter could be referred to Fig. 1

**Fig. 2:**
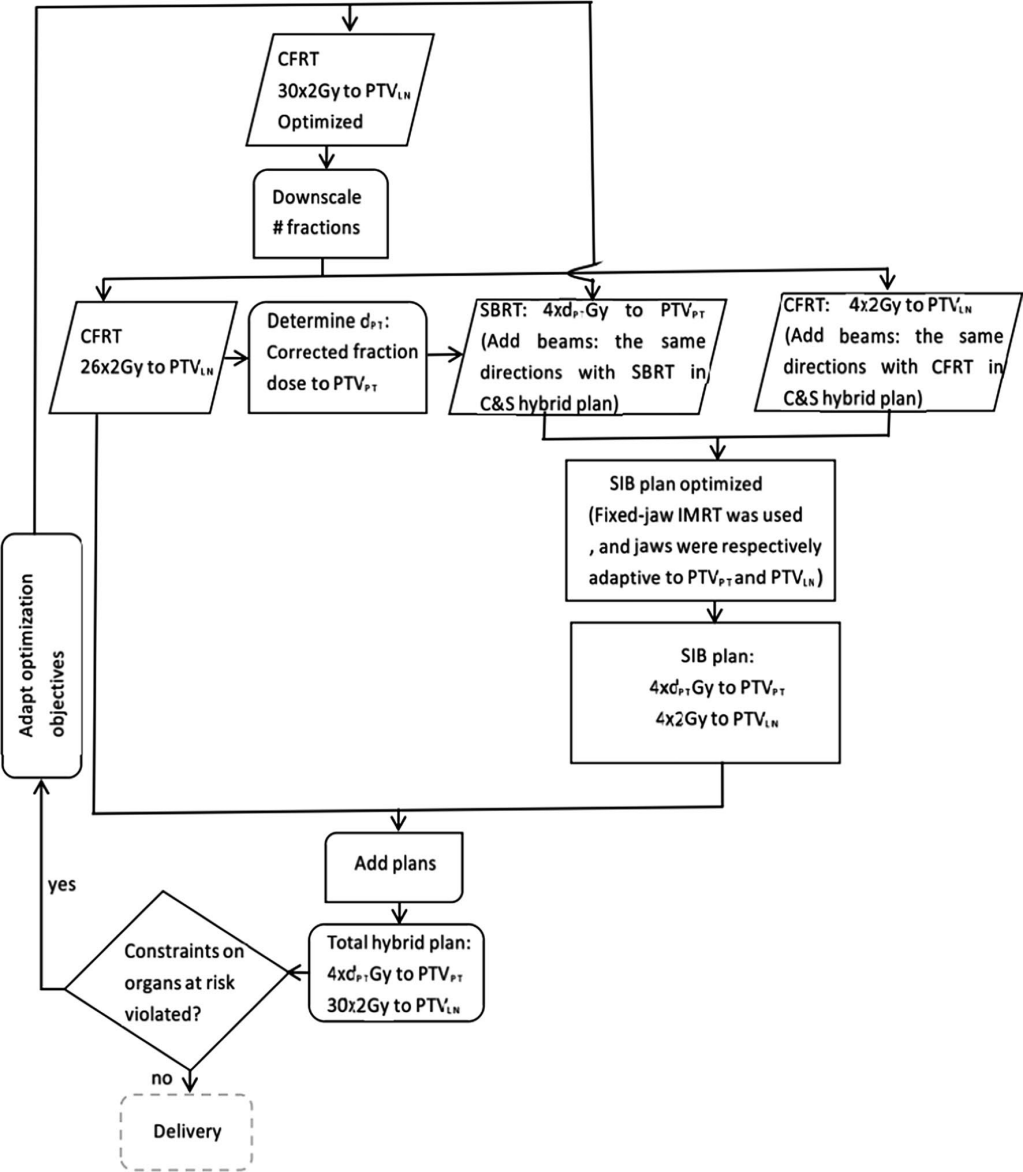
Workflow of C&SIB hybrid plan. PTV_LN_ = Planning Target Volume (PTV) of lymph nodes, PTV_PT_ = PTV of primary tumor, d_PT_ = fraction dose to primary tumor of SIB plan

### Treatment preparation

Each patient was fixed using a thermoplastic mask, and a 4D-planning CT scan ranged from the neck to the lower edge of the liver, including the entire lung, with 3-mm thickness was acquired. 10 respiratory phases CT images were transferred to MIM imaging system (MIM Software, Cleveland, USA). Average intensity projection CT (AIP-CT) and maximum intensity projection CT (MIP-CT) were acquired from MIM and transferred to the Pinnacle 9.10 treatment system (Philips, Fitchburg, USA).

For PTV of primary tumor (PTV_PT_) delineation, ITV was delineated on MIP-CT using lung window-level. MIP-CT was rigidly registered with the AIP-CT, and then ITV was copied to AIP-CT. ITV was expanded 5 mm uniformly to form PTV_PT_. The diagnostic FDG PET/CT was registered with AIP-CT for LN delineation. CTV of lymph node (CTV_LN_) was delineated on AIP-CT using mediastinal window-level, and expanded 5–10 mm to form PTV of lymph nodes (PTV_LN_). Appropriate adjustment of PTV_LN_ margin was made according to the actual motion of the patient. All contours were checked by a second dedicated radiation oncologist. AIP-CT was used for two kinds of hybrid plans.

### CFRT & SBRT boost plan (C&S plan)

All C&S hybrid plans were retrospectively completed by a senior physicist on first planning 4D-CT, including the three patients who received CFRT before SBRT boost. C&S hybrid plans were made by CFRT and SBRT boost plans. CFRT dose regime for LN was 2 Gy × 30f, and SBRT dose regimes for PT were ranged from 7.5 Gy × 8f to 12.5 Gy × 4f. For fair comparison, 12.5 Gy × 4f of SBRT dose regime was used for all hybrid plans in this study. All hybrid plans were made in Pinnacle 9.10 treatment planning system, and the selected linac for hybrid plans delivery was EDGE Linac (Varian, Palo Alto, USA).

All CFRT plans were made by 5–8 step-and-shoot IMRT fields with 6MV-X for PTV_LN_, and the planning method was presented in our previous work [13]. All plans were optimized by direct machine parameter optimize (DMPO) algorithm, and plan dose was calculated by Collapsed Cone Convolution Superposition (CCCs) algorithm built in planning system. The isocenter of the fields was located at the centroid of PTV_LN_, and the directions of the fields were adjusted to reduce the delivery to PTV_PT_. Dose volume histograms (DVHs) of total lung, spinal cord, heart and esophagus should be as low as possible to reserve space for the SBRT plans (Additional file 1), while 95% volume of PTV_LN_ was covered by the prescription dose.

SBRT boost plans were made by 9–11 coplanar IMRT fields for PTV_PT_, and it’s different from Haasbeek’s 8–12 non-coplanar 3D-conformal beams [14]. Each SBRT beam was optimized to produce a single segment, and the minimum segment aperture allowed was 3.5 cm, which corresponded to the projection of each individual beam’s-eye view of the PTV_PT_. This method offered a coverage of primary tumor volume in delivery and a steeper dose drop around PTV_PT_. The isocenter of SBRT fields was located at the centroid of PTV_PT_, and the directions of the fields were adjusted to reduce the delivery to PTV_LN_. The dose drop from the boundary of PTV_PT_ to 50% isodose line of the prescription dose had better be within 6 mm for SBRT plans. Unintended dose (D_99_) to PTV_PT_ obtained in CFRT plan was reduced from EQD_2_ corrected prescription dose of PTV_PT_, then the remaining EQD_2_ dose was converted to actual fraction dose of SBRT in proportion. The OARs dose constraints of SBRT plans should allow for the remaining space from CFRT plans.

### CFRT & SIB plan (C&SIB plan)

C&SIB hybrid plans were made from CFRT and sequential SIB plans, and the workflow of C&SIB was described in Fig. 2. CFRT dose regime for LN was 2 Gy × 26f, and sequential 2 Gy × 4f for LN was combined with 12.5 Gy × 4f simultaneous integrated boost for PT. CFRT plans of C&SIB were consistent with those of C&S planning. Directions and isocenters of sequential SIB beams were consistent with those of CFRT and SBRT in C&S planning. Fixed-jaw method was used to limit the jaw positions so that PTV_PT_ and PTV_LN_ could be covered by adaptive aperture of corresponding field. The method to calculate actual prescription dose of PTV_PT_ was the same with that of C&S planning, that allowed for intended dose obtained in 26 fractions of CFRT plans. The OARs dose constraints of SIB plans should allow for the remaining space from 26 fractions of CFRT plans (Additional file 1).

### Plan parameters

Physical plan parameters were derived from two kinds of hybrid plans in Pinnacle 9.10 system. Planning CT, RT structure, RT plan and RT dose of SBRT and SIB plans of each patient were then respectively imported into Matlab R2016a (The MathWorks Inc., MA, USA). SBRT and SIB plan dose were corrected by voxel-wise EQD_2_ [15] dose using an in-house Matlab script. EQD_2_ corrected SBRT and SIB dose were respectively superposed with 30 and 26 fractions physical plan dose of CFRT to acquire EQD_2_ plan parameters of C&S and C&SIB plans in Monaco planning system (Elekta, Stockhom, Sweden).

Physical plan parameters included: PTV_LN_ CI_60Gy_; PTV_(PT+LN)_ CI_60Gy_; V_20_ and mean lung dose (MLD) of total lung and ipsilateral lung; spinal cord D_max_; heart D_5_, D_30_, D_max_ and mean heart dose (MHD); esophagus D_max_ and V_50_. EQD_2_ plan parameters were the same with physical plan parameters except PTV_PT_ CI_140Gy_. The Conformity Index (CI) formula is CI = V^2^_RX_/(TV × V_RI_), where TV is the structure volume, V_RX_ is the structure volume covered by the Dose of Interest and V_RI_ is the total volume of the Dose of Interest. The Homogeneity Index (HI) formula is HI = D_5_/D_95_, where D_5_ is the dose covered 5% volume of the structure, and D_95_ is the dose covered 95% volume of the structure (Additional file 2).

### Regression models

There were four works included in this work: (1). Regression models were established between physical plan parameters and anatomical geometry features listed in Table 1. (2). Spearman’s correlation coefficients between physical and EQD_2_ plan parameters were calculated to verify the possibilities of regression models of EQD_2_ plan parameters. Models were also tried to establish between EQD_2_ plan parameters and anatomical geometry features. (3). The consistence of coefficients of determination (*R*^*2*^) of regression models between physical and EQD_2_ plan parameters was determined. Its correlation to spearman’s correlation coefficients (*r*) between physical and EQD_2_ plan parameters for two kinds of hybrid plans was investigated. (4). Physical and EQD_2_ plan parameters were respectively compared with models predictions to verify the effectiveness of the models by two new patients.

### Data analysis

Regression models of plan parameters were established by SPSS 20 statistical software (IBM, USA). It showed high goodness of model fitting when *R*^*2*^(Coefficients of determination) ≥ 0.7, moderate goodness of model fitting when 0.7 > *R*^*2*^ ≥ 0.5, and poor goodness of model fitting when *R*^*2*^ < 0.5. Spearman’s correlation analysis between physical and EQD_2_ plan parameters was also performed using SPSS 20 statistical software. Physical and EQD_2_ parameters showed high correlations when *r* ≥ 0.8; and moderate correlations when 0.8 > *r* ≥ 0.6.

## Results

### Regression models of physical plan parameters

For physical plan parameters, we established regression models between physical plan parameters and anatomical geometry features for two kinds of hybrid plans. PTV_LN_ CI_60Gy_ showed a high linear correlation with PTV_PT_ volume, PTV_LN_ volume, and min distance_(PT to LN)_ for both kinds of hybrid plans (*R*^*2*^ = 0.811, *P* < 0.001 for C&S plans, and *R*^*2*^ = 0.779, *P* < 0.001 for C&SIB plans).

PTV_(PT+LN)_ CI_60Gy_ showed a high linear correlation with PTV_PT_ volume, PTV_LN_ volume, min distance_(PT to LN)_, and overlapping slices thickness_(PT and LN)_ (*R*^*2*^ = 0.820, *P* < 0.001 for C&S plans, and *R*^*2*^ = 0.833, *P* < 0.001 for C&SIB plans).

**Fig. 3:**
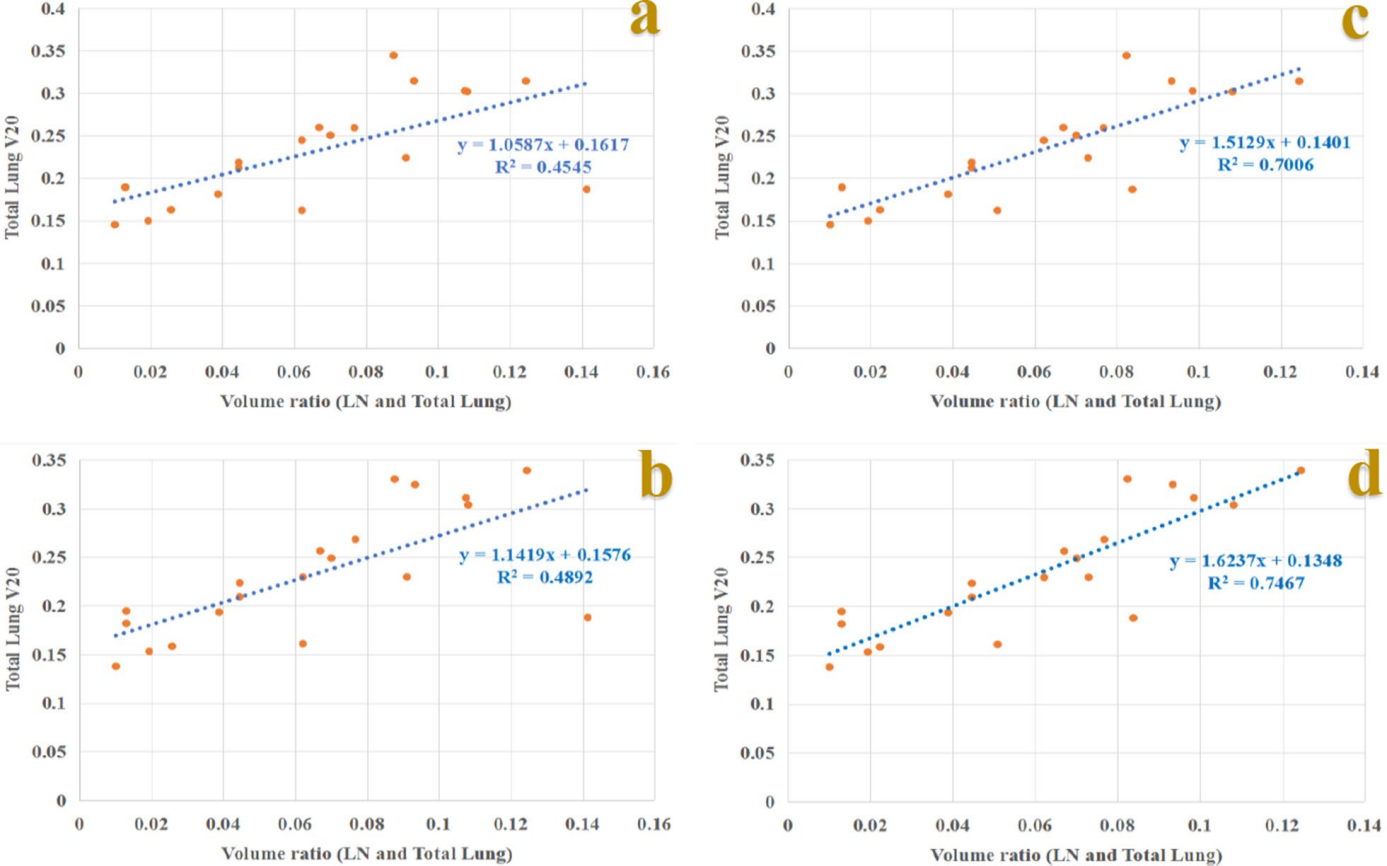
Regression models between physical total lung V_20_ and volume ratio_(LN to total lung)_ for C&S and C&SIB plans (**a**, **b**); and models between physical total lung V_20_ and corrected volume ratio_(LN to total lung)_ for C&S and C&SIB plans (**c**, **d**)

V_20_ (Fig. 3a, b) and MLD of total lung showed poor linear correlations with volume ratio_(LN to total lung)_ (*R*^*2*^ = 0.455, *P* < 0.001 and *R*^*2*^ = 0.450, *P* < 0.001 for C&S plans; *R*^*2*^ = 0.489, *P* < 0.001 and *R*^*2*^ = 0.487, *P* < 0.001 for C&SIB plans). When neck lymph node metastasis was excluded from PTV_LN_ volume, V_20_ (Fig. 3c, d) and MLD of total lung showed high linear correlations with corrected volume ratio_(LN to total lung)_ (*R*^*2*^ = 0.701, *P* < 0.001 and *R*^*2*^ = 0.703, *P* < 0.001 for C&S plans; *R*^*2*^ = 0.747, *P* < 0.001 and *R*^*2*^ = 0.730, *P* < 0.001 for C&SIB plans).

V_20_ and MLD of ipsilateral lung showed moderate linear correlations with min distance_(PT to LN)_, volume ratio_(PT to ipsilateral lung)_, and corrected volume ratio_(LN to ipsilateral lung)_.

When heart was partially overlapped with PTV_LN_, heart D_30_ and MHD showed high correlations of S type function with volume ratio of overlapping structure_(heart and LN)_ to PTV_LN_ (R_HLL_) (Heart D_30_ = e^8.11–0.024/RHLL^, *R*^*2*^ = 0.767, *P* < 0.001; MHD = e^7.677–0.017/RHLL^, *R*^*2*^ = 0.708, *P* < 0.001 for C&S plans, and heart D_30_ = e^8.098–0.024/RHLL^, *R*^*2*^ = 0.775, *P* < 0.001; MHD = e^7.656–0.017/RHLL^, *R*^*2*^ = 0.700, *P* < 0.001 for C&SIB plans). Heart D_5_ showed a moderate correlation of S function with volume ratio of overlapping structure_(heart and LN)_ to PTV_LN_.

When esophagus was partially overlapped with PTV_LN_, physical esophagus D_max_ showed a moderate inverse function with min distance_(PTVPT to ESO)_ for C&SIB plans (*R*^*2*^=0.692, *P* < 0.001). Esophagus V_50_ had a moderate correlation of cubic function with volume ratio of overlapping structure_(ESO and LN)_ to ESO (*R*^*2*^ = 0.648, *P* = 0.005 for C&S plans; and *R*^*2*^ = 0.695, *P* = 0.002 for C&SIB plans).

### Regression models of EQD_2_ plan parameters

**Fig. 4:**
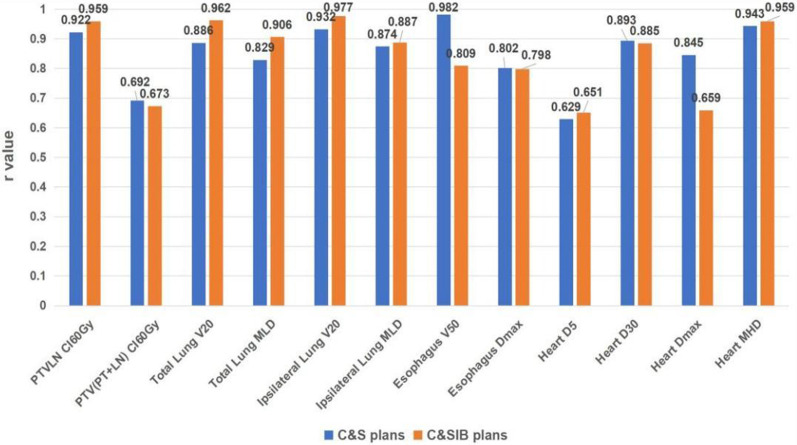
Spearman’s correlation coefficients between physical and EQD_2_ plan parameters for two kinds of hybrid plans

Possibilities of regression models of EQD_2_ plan parameters were investigated by the correlations between physical and EQD_2_ plan parameters. Spearman’s correlation coefficients were calculated between physical and EQD_2_ plan parameters, and the correlations were statistically significant for all plan parameters. Physical and EQD_2_ parameters showed high correlations (*r* ≥ 0.8) for PTV_LN_ CI_60Gy_, total lung V_20_ and MLD, ipsilateral lung V_20_ and MLD, esophagus V_50_ and D_max_, heart D_30_ and MHD (Fig. 4). Moderate correlations (0.8 > *r* ≥ 0.6) were shown for PTV_(PT+LN)_ CI_60Gy_, heart D_5_ and D_max_.

For EQD_2_ plan parameters, regression models were also established for two kinds of hybrid plans. PTV_LN_ CI_60Gy_ showed a high linear correlation with PTV_PT_ volume, PTV_LN_ volume, and min distance_(PT to LN)_ for both kinds of hybrid plans (*R*^*2*^ = 0.85, *P* < 0.001 for C&S plans, and *R*^*2*^ = 0.812, *P* < 0.001 for C&SIB plans).

PTV_(PT+LN)_ CI_60Gy_ showed a linear correlation with PTV_PT_ volume, PTV_LN_ volume, min distance_(PT to LN)_, and overlapping slices thickness_(PT and LN)_ (*R*^*2*^ = 0.845, *P* < 0.001 for C&S plans, and *R*^*2*^ = 0.668, *P* = 0.002 for C&SIB plans).

**Fig. 5:**
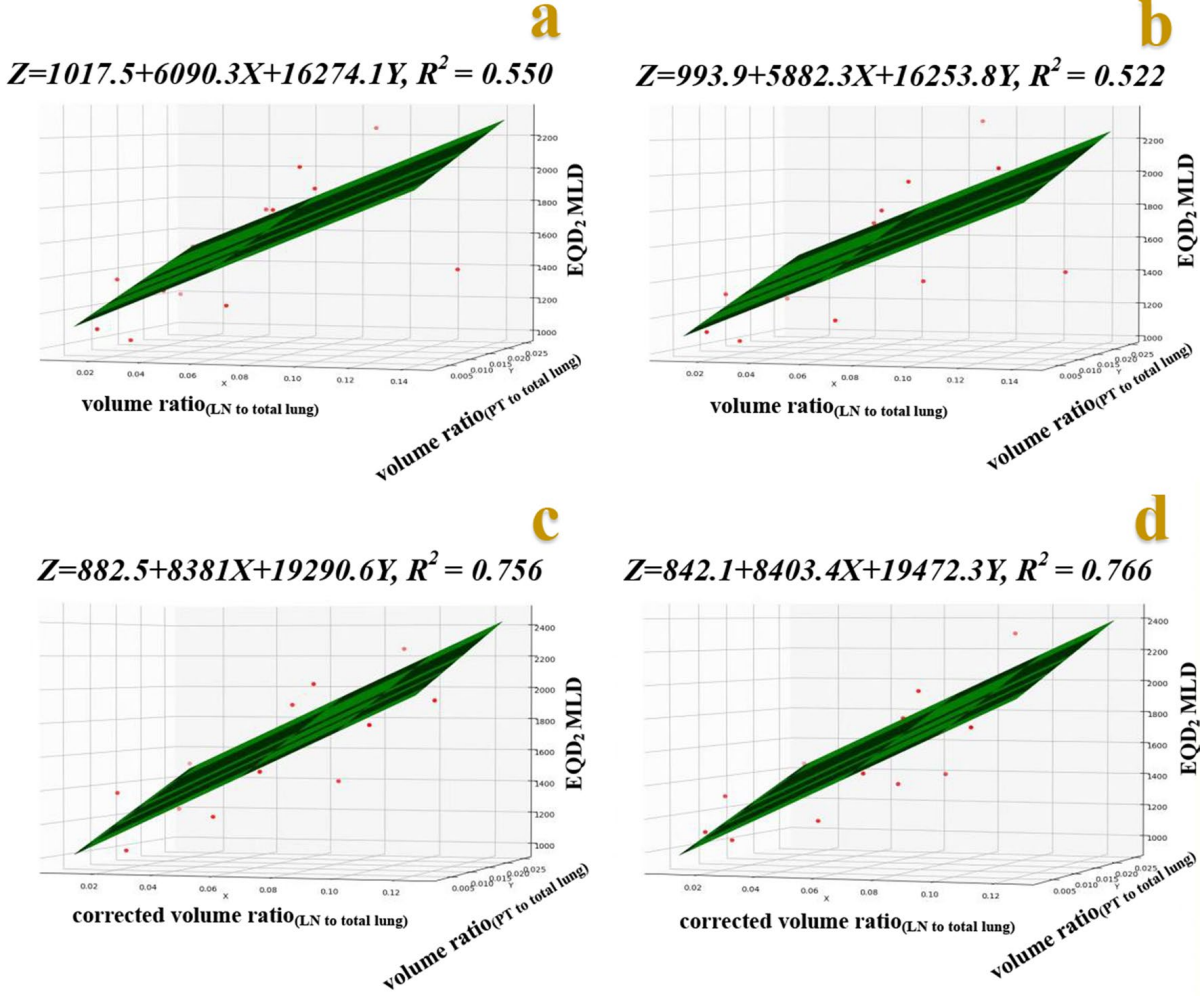
Regression models between EQD_2_ total lung MLD and volume ratio_(LN to total lung)_ combined with volume ratio_(PT to total lung)_ for C&S and C&SIB plans (**a**, **b**); regression models between EQD_2_ total lung MLD and corrected volume ratio_(LN to total lung)_ combined with volume ratio_(PT to total lung)_ for C&S and C&SIB plans (**c**, **d**). *Z: EQD*_2_ total lung MLD; X: volume ratio_(LN to total lung)_ (**a**, **b**), and corrected volume ratio_(LN to total lung)_(**c**, **d**); Y: volume ratio_(PT to total lung)_

Total lung MLD showed a linear correlation with volume ratio_(LN to total lung)_ and volume ratio_(PT to total lung)_ (Fig. 5a, b) (*R*^*2*^ = 0.550, *P* = 0.001 for C&S plans; *R*^*2*^ = 0.522, *P* = 0.002 for C&SIB plans). When neck lymph node metastasis was excluded from PTV_LN_ volume, total lung MLD showed a high linear correlation with corrected volume ratio_(LN to total lung)_ and volume ratio_(PT to total lung)_ (Fig. 5c, d) (*R*^*2*^ = 0.756, *P* < 0.001 for C&S plans; *R*^*2*^ = 0.766, *P* < 0.001 for C&SIB plans).

Total lung V_20_ showed a linear correlation with corrected volume ratio_(LN to total lung)_ (*R*^*2*^ = 0.663, *P* < 0.001 for C&S plans; *R*^*2*^ = 0.705, *P* < 0.001 for C&SIB plans).

V_20_ and MLD of ipsilateral lung respectively showed moderate and high linear correlations with three geometry features of min distance_(PT to LN)_, volume ratio_(PT to ipsilateral lung)_, and corrected volume ratio_(LN to ipsilateral lung)_.

When heart was partially overlapped with PTV_LN_, heart D_5_ showed a high correlation of S type function with volume ratio of overlapping structure_(heart and LN)_ to heart (*R*^*2*^ = 0.867, *P* < 0.001 for C&S plans, and *R*^*2*^ = 0.842, *P* < 0.001 for C&SIB plans). Heart D_30_ had a moderate correlation of S type function with volume ratio of overlapping structure_(heart and LN)_ to PTV_LN_, while MHD had a moderate correlation of S type function with volume ratio of overlapping structure_(heart and LN)_ to heart. Heart D_max_ showed a moderate correlation of inverse function with min distance_(PTVPT to heart)_ (*R*^*2*^= 0.573, *P* = 0.031 for C&S plans; and *R*^*2*^= 0.621, *P* = 0.001 for C&SIB plans).

Esophagus D_max_ had a moderate correlation of inverse function with min distance_(PTVPT to ESO)_.

### Fitting goodness of regression models

**Fig.6:**
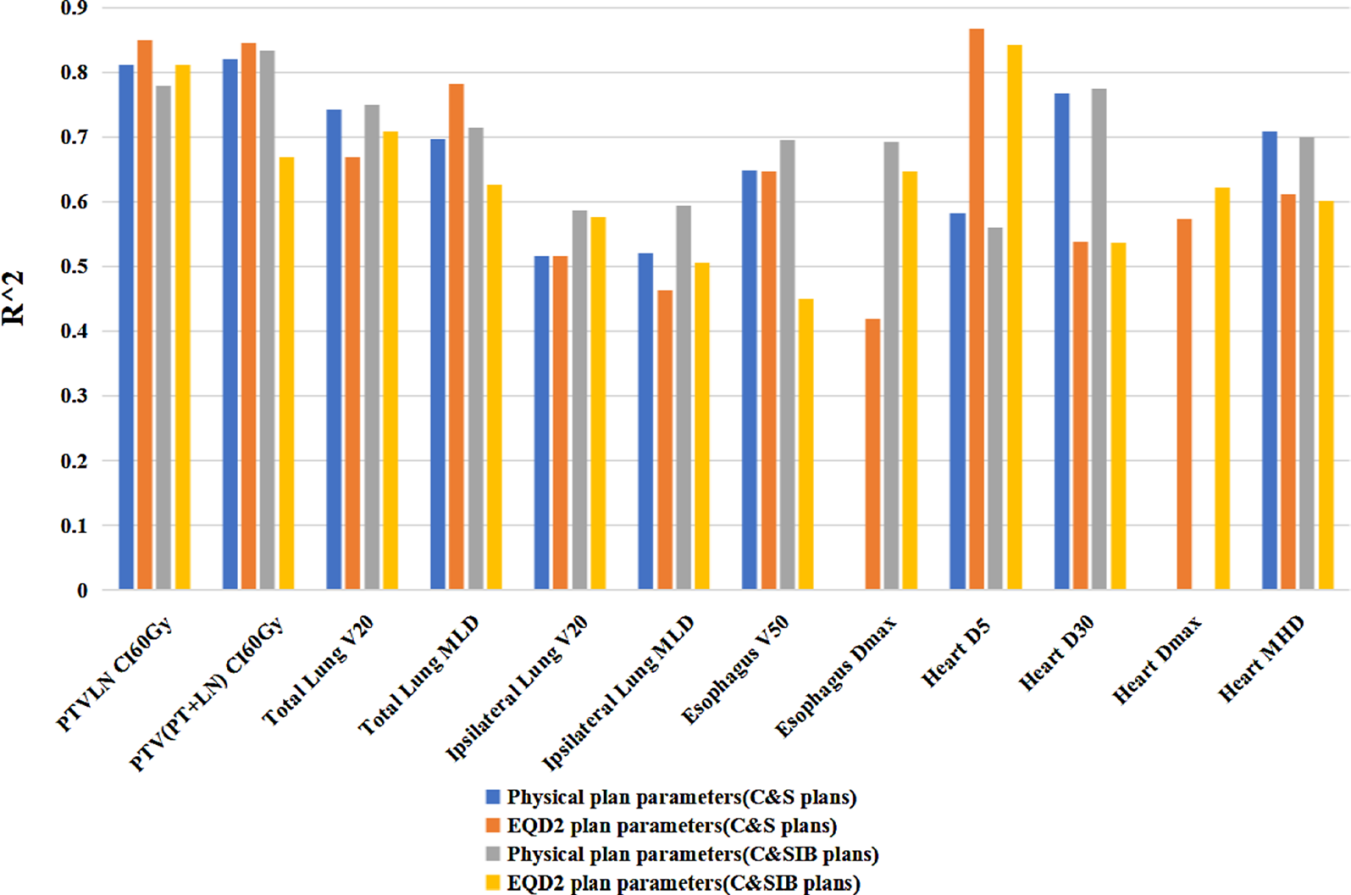
Coefficients of determination (*R*^*2*^) for regression models between plan parameters listed in the X-axis and geometric parameters of the anatomy. Regression models were described by using the following format: *Plan parameter(geometric parameters of the anatomy): correlation type. PTV*_*LN*_* CI*_*60Gy*_* (PTV*_*PT*_* volume, PTV*_*LN*_* volume, and min distance*_*(PT to LN)*_*): Linear correlation: PTV*_*(PT*+*LN)*_* CI*_*60Gy*_* (PTV*_*PT*_* volume, PTV*_*LN*_* volume, min distance*_*(PT to LN)*_*, and overlapping slices thickness*_*(PT and LN)*_*): Linear correlation. Total lung V*_*20*_* (corrected volume ratio*_*(LN to total lung)*_*): Linear correlation: Physical total lung MLD (corrected volume ratio*_*(LN to total lung)*_*): Linear correlation: EQD*_*2*_* total lung MLD (corrected volume ratio*_*(LN to total lung)*_*, volume ratio*_*(PT to total lung)*_*): Linear correlation: Ipsilateral lung V*_*20*_* and MLD (min distance*_*(PT to LN)*_*, volume ratio*_*(PT to ipsilateral lung)*_*, and corrected volume ratio*_*(LN to ipsilateral lung)*_*): Linear correlation: Heart D*_*30*_* (volume ratio of overlapping structure*_*(heart and LN)*_* to PTV*_*LN*_*): S type function. Physical heart D*_*5*_* and MHD (volume ratio of overlapping structure*_*(heart and LN)*_* to PTV*_*LN*_*): S type function. EQD*_*2*_* heart D*_*5*_* and MHD (volume ratio of overlapping structure*_*(heart and LN)*_* to Heart): S type function. EQD*_*2*_* heart D*_*max*_* (min distance *_*(PTVPT to heart)*_*): Inverse function. Physical esophagus D*_*max*_* (min distance*_*(PTVPT to ESO)*_*): Inverse function for C&SIB plans. EQD*_*2*_* esophagus D*_*max*_* (min distance*_*(PTVPT to ESO)*_*): Inverse function*

Coefficients of determination (*R*^*2*^) of regression models of physical and EQD_2_ plan parameters for two kinds of hybrid plans were shown in Fig. 6.

For the parameters of PTV_LN_ CI_60Gy_, total lung V_20_ and MLD, ipsilateral lung MLD, heart D_5_ and MHD, it indicated that the consistence of the fitting goodness of regression models between physical and EQD_2_ plan parameters of C&SIB plans was better than that of C&S plans, while the correlations between physical and EQD_2_ plan parameters of C&SIB plans were higher than those of C&S plans (Fig. 4).

For the parameters of PTV_(PT+LN)_ CI_60Gy_, esophagus V_50_, and heart D_5_, the consistence of the fitting goodness of regression models between physical and EQD_2_ plan parameters of C&SIB plans was poorer than that of C&S plans, while the correlations of C&SIB plans were lower than those of C&S plans.

As the correlation between physical heart D_max_ and min distance_(PTVPT to heart)_ couldn’t been established in this work, meanwhile, the correlation between physical esophagus D_max_ and min distance_(PTVPT to ESO)_ couldn’t been established for C&S plans, we didn’t show these results in Fig. 6.

### Effectiveness of regression models

**Fig. 7:**
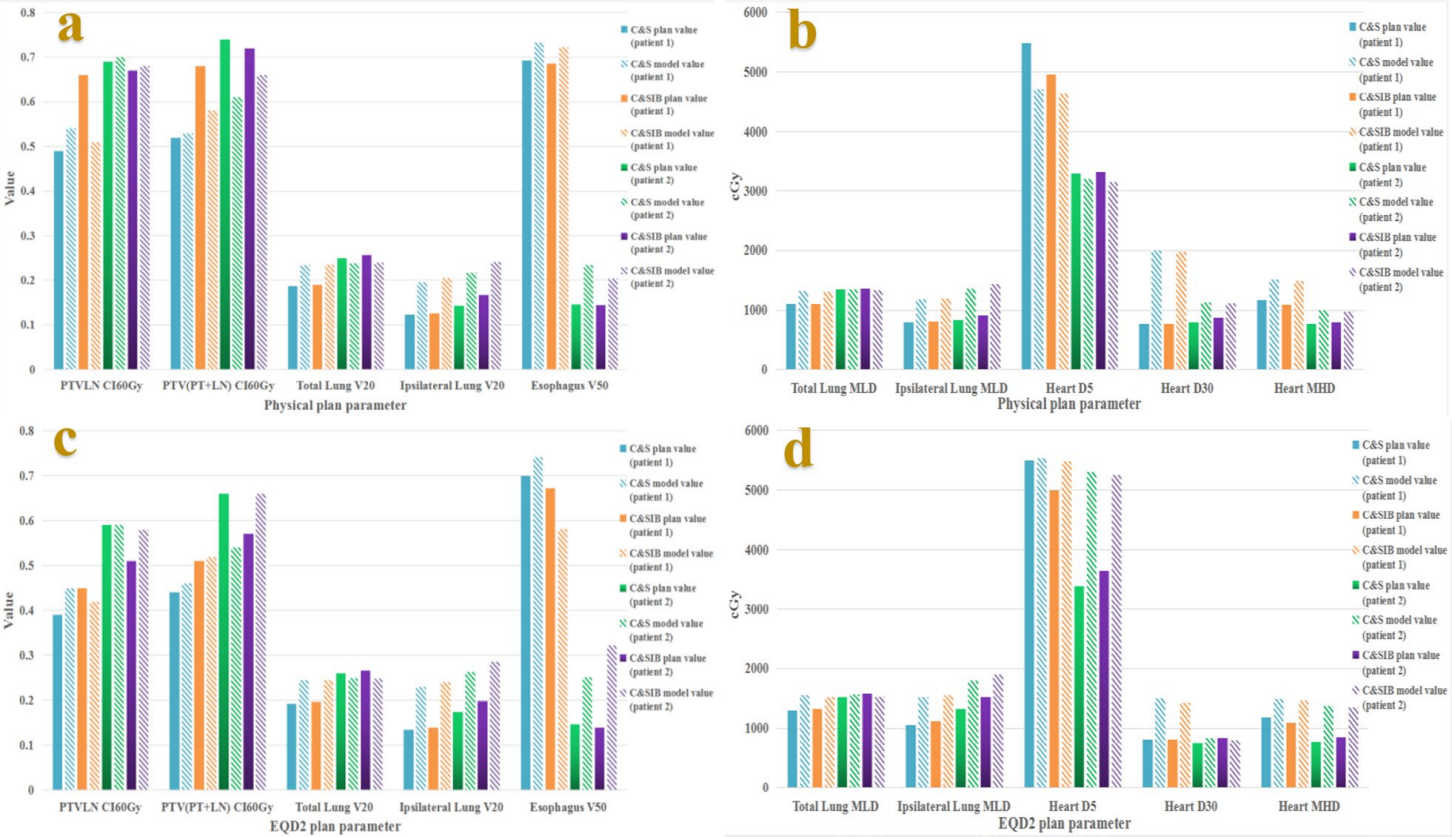
Physical models predictions were compared with physical plan parameters for two kinds of hybrid plans by two new patients (**a**, **b**), EQD_2_ models predictions were compared with EQD_2_ plan parameters for two kinds of hybrid plans by two new patients (**c**, **d**)

Figure 7 shows the performance of the models of physical and EQD_2_ plan parameters for two kinds of hybrid plans.

It indicated that physical models predictions of PTV_LN_ CI_60Gy_, total lung V_20_, total lung MLD, and heart D_5_ had good consistence with physical plan parameters for second patient (Fig. 7a, b). Meanwhile, for the physical plan parameters of total lung V_20_, ipsilateral lung V_20_, ipsilateral lung MLD, and esophagus V_50_, physical models could predict that which kind of hybrid plan was high than the other one for two new patients.

EQD_2_ models predictions of total lung V_20_, total lung MLD, and heart D_30_ had good consistence with EQD_2_ plan parameters for the second patient (Fig. 7c, d). Meanwhile, for EQD_2_ plan parameters of total lung V_20_, ipsilateral lung V_20_, and ipsilateral lung MLD, EQD_2_ models could predict that which kind of hybrid plan was high than the other one for two new patients.

## Discussion

For stage III NSCLC with solitary lung lesion and lymph node metastasis, regression models of physical and EQD_2_ plan parameters were established based on anatomical geometry features for two kinds of hybrid plans in this study, and regression models had at least moderate fitting goodness. The possibilities of regression models of EQD_2_ plan parameters were verified by the correlations between physical and EQD_2_ plan parameters. The consistence of the fitting goodness of regression models between physical and EQD_2_ plan parameters had a positive correlation with spearman’s correlation coefficients between physical and EQD_2_ plan parameters for two kinds of hybrid plans. For physical and EQD_2_ parameters of total lung V_20_, ipsilateral lung V_20_, and ipsilateral lung MLD, the models could predict that which kind of hybrid plan was high than the other one for two new patients. PT of all hybrid plans were given a prescription dose of 4 × 12.5 Gy in this study.

At present, different strategies could be used for hybrid planning [7–12], and we chose two main methods in this work. C&S hybrid planning is simple and time-saving, but the interaction effect of hybrid plan components (CFRT and SBRT) is significant when the two lesions are close. C&SIB hybrid planning is complex and time-consuming. The first step of C&SIB planning (CFRT) to irradiate LN still has an impact on PT dose, although in the second step, the interaction effect could be reduced to a certain extent in the SIB plan optimization. Due to the different dose schemes of hybrid plan components (CFRT, SBRT and SIB), it would be more appropriate to convert the physical dose of the latter two plans to EQD_2_ corrected dose in evaluation process of hybrid plans.

It’s still not very clear which kind of hybrid planning has more dosimetric advantages, especially EQD_2_ dose advantages when the patients have different anatomical geometry features. It would be indeterminate in choosing hybrid planning strategy under the above situation. Regression models of physical and EQD_2_ plan parameters would be effective and time saving tools to solve such problems.

There have been few studies about the models for predicting physical plan parameters, especially EQD_2_ plan parameters of two kinds of hybrid plans to our knowledge. The interaction effect of each kind of hybrid planning may be variously related to the geometry features, which provided us with inspiration and opportunity to find the optimal method of hybrid planning. This is the first research to assess the potential of regression models of physical and EQD_2_ plan parameters for two methods of hybrid planning. Regression models of EQD_2_ plan parameters proposed in this work were based on the assumption that we have used the appropriate EQD_2_ calculation model for SBRT and SIB plans [15], and the correct target and OARs α/β values [16].

For the three patients who received CFRT before SBRT boost in this work, the generation of a summed up plan was even more difficult due to the presence of two planning CTs and different geometries of the anatomy and the tumor, hybrid plans were retrospectively made for these three patients the same as the other seventeen patients on the first planning 4D-CT. This would be suitable for the reason that the main purpose of this work is to propose the concept of plan parameters prediction models of two kinds of hybrid plans, while ignoring the changes of anatomical structure of the three patients during two planning CTs.

In this work, physical and EQD_2_ plan parameters of PTV_LN_ CI_60Gy_ have shown strong positive correlations with PTV_LN_ volume and min distance_(PT to LN)_, and strong negative correlations with PTV_PT_ volume for two kinds of hybrid plans. PTV_(PT+LN)_ CI_60Gy_ is not only correlated with above three geometry features, but also negatively correlated with overlapping slices thickness_(PT and LN)_. It could be inferred that interaction effect between two lesions is mainly affected by four features above.

PTV/lung volume ratio has been shown a reliable factor for predicting lung dose [17, 18]. In this study, as 6 out of 20 patients had neck lymph node metastasis, physical and EQD_2_ total lung V_20_ had poor linear correlations with volume ratio_(LN to total lung)_. It may be easy to understand that lung dose was almost unaffected by the volume of neck lymph node metastasis_._ When neck lymph node metastasis was excluded from PTV_LN_ volume, physical and EQD_2_ total lung V_20_ showed a high linear correlation with corrected volume ratio_(LN to total lung)._ Meanwhile, physical total lung MLD had a high linear correlation with corrected volume ratio_(LN to total lung)_, while EQD_2_ total lung MLD was not only affected by corrected volume ratio_(LN to total lung)_ but also volume ratio_(PT to total lung)._ It can be explained that EQD_2_ lung dose around PTV_PT_ was nearly 3.1 times than physical lung dose (EQD_2_ dose = D(d + α/β)/(2 + α/β), D: total dose (50 Gy); d: fraction dose (12.5 Gy); lung α/β = 3) [15, 16], while PTV_PT_ volumes were much smaller than PTV_LN_ volumes. As a result, the presence of PTV_PT_ didn’t significantly affect physical and EQD_2_ total lung volume dose, such as V_20_, but could affect EQD_2_ total lung MLD.

It also revealed that heart D_5_, D_30_ and MHD would be more susceptible to overlapping structure_(heart and LN)_. Another interesting result was that min distance_(PT to ESO)_ may be an important feature for predicting EQD_2_ esophageal max dose for hybrid plans. It indicated that hybrid plans components (SBRT or SIB) may also have an important influence on EQD_2_ esophageal max dose, though we may pay more attention on the influence of CFRT plans.

A similar knowledge-based planning solution called RapidPlan [19, 20] takes a library of precious treatment plans and uses regression analysis to determine correlations between geometric and dosimetric features of the planning target volumes (PTVs) and OARs of the library plans. This method forms the model, and the model is then used to predict a range of achievable plan parameters for a prospective patient. For stage III NSCLC patients, it’s challenging to predict the plan parameters of hybrid plans when PT received SBRT with different dose regimens and LN received CFRT. Physical and EQD_2_ plan parameters derived from regression models could be used as benchmarking protocols of hybrid planning in routine practice with the models improved.

Under the assumption that EQD_2_ dose calculation model is reliable (it’s not the scope of this work), we've made sure that there were connections between physical and EQD_2_ plan parameters, and it’s feasible to establish the regression models of EQD_2_ plan parameters. We could see that the regression models had at least moderate goodness in this work. Meanwhile, the consistence of the fitting goodness of regression models between physical and EQD_2_ plan parameters had a positive correlation with spearman’s correlation coefficients between physical and EQD_2_ plan parameters for two kinds of hybrid plans.

One of the most important purposes of this study was to investigate if regression models could exactly predict which kind of hybrid plan was superior to the other one for physical and EQD_2_ plan parameters. As the patients selection was really hard, and the quantity of patients suitable for radiotherapy with hybrid plan was limited in this work, we could use only two new patients data in the models validation process. And frankly, this is a proof of concept which may work or not in the end when all of the variabilities in patient anatomies and anatomy of the disease itself were considered. As a pilot study, we thought it would be acceptable to some extent even if a statistically significant validation hasn’t been done. From the validation results of two patients, we could preliminarily evaluate the effectiveness of the regression models.

For total lung V_20_, ipsilateral lung V_20_, and ipsilateral lung MLD, the models could predict that C&SIB plans were higher than C&S plans for two new patients. In this work, physical and EQD_2_ models were based on 20 patients data including 4 patients whose mediastinal lymph node and primary lesions were located in left and right lobes respectively. As two new patients by whom we verified the models were exactly such patients, we could see that only several physical and EQD_2_ models predictions, such as total lung V_20_ and MLD, had good consistence with plan parameters for the second patient. This situation indicated that more patients needed to be used in the models fitting.

There were still some limitations in this work. First, lung cancer patients suitable for hybrid planning were highly selected. Most patients with stage III NSCLC have large central lung lesions, only approximately 5–10% of patients are suitable for hybrid planning. This number would likely increase in case of SBRT dose regime with more fractions. Secondly, the relationship between geometric features and physical/EQD_2_ plan parameters derived from the 20 patients wasn’t sufficient to cover the complete connection. As the patients increase, smarter models could be developed to predict the performance of different hybrid planning for stage III NSCLC.

In conclusion, regression models of physical and EQD_2_ plan parameters were established for two kinds of hybrid planning, and the regression models had at least moderate goodness. The possibilities of regression models of EQD_2_ plan parameters were verified by the correlations between physical and EQD_2_ plan parameters, and the consistence of the fitting goodness of regression models between physical and EQD_2_ plan parameters had a positive correlation with spearman’s correlation coefficients between physical and EQD_2_ plan parameters. The models have a potential to predict physical and EQD_2_ plan parameters for two kinds of hybrid planning. The models could be gradually developed into an automatic optimization method for hybrid planning based on prior knowledge.

## Supplementary Information

**Additional file 1.** Plan objectives for two kinds of hybrid planning.

**Additional file 2.** Physical and EQD2 plan parameters of two kinds of hybrid plans.

## Data Availability

The datasets used and/or analyzed during the current study are available from the corresponding author on reasonable request.

## Abbreviations

EQD_2_: Equivalent Dose in 2 Gy/f
NSCLC: None-small cell lung cancer
CFRT: Conventional fraction radiotherapy
SBRT: Stereotactic Body Radiation Therapy
SIB: Simultaneous integrated boost
C&S plan: CFRT&SBRT plan
C&SIB plan: CFRT&SIB plan
DRR: Digitally reconstructured radiograph
PT: Primary tumor
LN: Lymph node
PTV_PT_: PTV of PT
PTV_LN_: PTV of LN
ITV: Internal target volume
ESO: Esophagus
R_HLL_: Volume ratio of overlapping structure_(heart and LN)_ to PTV_LN_
R_HLH_: Volume ratio of overlapping structure_(heart and LN)_ to heart
MLD: Mean lung dose
MHD: Mean heart dose
CI: Conformity Index
HI: Homogeneity Index
