# Supplementary material for: Regression models for predicting physical and EQD2 plan parameters of two methods of hybrid planning for stage III NSCLC
Source: Radiat Oncol. 2021 Jun 27;16:119. doi: 10.1186/s13014-021-01848-9 (PMC8237456; doi:10.1186/s13014-021-01848-9)
Supplement: Supplementary file 1 — Additional file 1. Plan objectives for two kinds of hybrid planning. [file 13014_2021_1848_MOESM1_ESM.docx]

| **Organ** | **Parameters** | **Constraints** | **Acceptable** |
| --- | --- | --- | --- |
| PTV_PT_ | V_100%_ | ≥ 95% | ≥ 93% |
| PTV_LN_ | V_90%_ | ≥ 99% | ≥ 97% |
| Total Lung | V_5_ | ≤ 60% | ≤ 65% |
|  | V_20_ | ≤ 30% | ≤ 35% |
|  | MLD | ≤ 20Gy | ≤ 23Gy |
| Ipsilateral Lung | V_5_ | ≤ 70% | ≤ 75% |
|  | V_20_ | ≤ 45% | ≤ 50% |
|  | MLD | ≤ 23Gy | ≤ 28Gy |
| Spinal cord | D_max_ | ≤ 50Gy | ≤ 50Gy |
| Esophagus | V_35_ | ≤ 65% | ≤ 70% |
|  | V_50_ | ≤ 50% | ≤ 55% |
|  | D_max_ | ≤ 66Gy | ≤ 66Gy |
| Heart | D_5_ | ≤ 60% | ≤ 65% |
|  | D_30_ | ≤ 30% | ≤ 35% |
|  | D_max_ | ≤ 66Gy | ≤ 80Gy |
|  | MHD | ≤ 25Gy | ≤ 30Gy |

**Tab.2 Hybrid plans objectives**

**Note: plan objectives were desired for the superimposed physical dose of two components of hybrid plans.**

**Tab.3 CFRT plan constraints prioritization orders**

| **Organ** | **Parameter** | **Constraints** | **Weight** |
| --- | --- | --- | --- |
| PTV_LN_ | V_100%_ | ≥ 95% | 100 |
|  | V_95%_ | ≥ 100% | 100 |
|  | D_max_ | ≤ 65Gy | 90 |
| Total Lung | V_5_ | ≤ 45% | 85 |
|  | V_20_ | ≤ 23% | 95 |
| Ipsilateral Lung | V_5_ | ≤ 50% | 85 |
|  | V_20_ | ≤ 35% | 85 |
| Spinal cord | D_max_ | ≤ 44Gy | 95 |
| Esophagus | V_35_ | ≤ 60% | 80 |
|  | V_50_ | ≤ 45% | 85 |
|  | D_max_ | ≤ 65Gy | 95 |
| Heart | D_5_ | ≤ 55% | 85 |
|  | D_30_ | ≤ 25% | 80 |
|  | D_max_ | ≤ 63Gy | 75 |
| Ring 6 | D_max_ | ≤ 54Gy | 80 |
| Ring 12 | D_max_ | ≤ 48Gy | 75 |
| Ring 18 | D_max_ | ≤ 42Gy | 70 |

**Note:**

1. **weight values were set as the objectives prioritization orders in Pinnacle 9.10 treatment planning system.**
2. **Ring 6: a dose auxiliary ring that was made by BODY structure subtracting the external expansion of 6 mm from PTV_LN_.**
3. **Ring 12: a dose auxiliary ring that was made by BODY structure subtracting the external expansion of 12 mm from PTV_LN_.**
4. **Ring 18: a dose auxiliary ring that was made by BODY structure subtracting the external expansion of 18 mm from PTV_LN_.**

**Tab.4 SBRT plan constraints prioritization orders**

| **Organ** | **Parameter** | **Constraints** | **Weight** |
| --- | --- | --- | --- |
| PTV_PT_ | V_100%_ | ≥ 95% | 100 |
|  | V_95%_ | ≥ 100% | 100 |
| Total Lung | V_5_ | ≤ 15% | 90 |
|  | V_20_ | ≤ 8% | 90 |
| Ipsilateral Lung | V_5_ | ≤ 20% | 85 |
|  | V_20_ | ≤ 15% | 85 |
| Spinal cord | D_max_ | ≤ 5Gy | 90 |
| R6 | D_max_ | ≤ 24Gy | 85 |
| R12 | D_max_ | ≤ 18Gy | 80 |
| R18 | D_max_ | ≤ 12Gy | 75 |

**Note:**

1. **weight values were set as the objectives prioritization orders in Pinnacle 9.10 treatment planning system.**
2. **R6: a dose auxiliary ring that was made by BODY structure subtracting the external expansion of 6 mm from PTV_PT_.**
3. **R12: a dose auxiliary ring that was made by BODY structure subtracting the external expansion of 12 mm from PTV_PT_.**
4. **R18: a dose auxiliary ring that was made by BODY structure subtracting the external expansion of 18 mm from PTV_PT_.**

**Tab.5 SIB plan constraints prioritization orders**

| **Organ** | **Parameter** | **Constraints** | **Weight** |
| --- | --- | --- | --- |
| PTV_LN_ | V_8Gy_ | ≥ 95% | 100 |
|  | V_7.6Gy_ | ≥ 100% | 100 |
|  | D_max_ | ≤ 8.8Gy | 85 |
| PTV_PT_ | V_50Gy_ | ≥ 95% | 100 |
|  | V_47.5Gy_ | ≥ 100% | 100 |
| Total Lung | V_5_ | ≤ 25% | 85 |
|  | V_20_ | ≤ 15% | 95 |
| Ipsilateral Lung | V_5_ | ≤ 30% | 85 |
|  | V_20_ | ≤ 20% | 85 |
| Spinal cord | D_max_ | ≤ 8Gy | 95 |
| Esophagus | D_max_ | ≤ 8.8Gy | 90 |
| Heart | D_5_ | ≤ 5% | 85 |
|  | D_max_ | ≤ 8.8Gy | 75 |
| Ring 6cm | D_max_ | ≤ 30Gy | 85 |
| Ring 12cm | D_max_ | ≤ 26Gy | 80 |
| R 6cm | D_max_ | ≤ 7Gy | 85 |
| R 12cm | D_max_ | ≤ 6Gy | 80 |

**Note:**

1. **weight values were set as the objectives prioritization orders in Pinnacle 9.10 treatment planning system.**
2. **Ring 6cm: a dose auxiliary ring that was made by the external expansion of 6 mm from PTV_PT_ subtracting PTV_PT_.**
3. **Ring 12cm: a dose auxiliary ring that was made by the external expansion of 12 mm from PTV_PT_ subtracting the external expansion of 6 mm from PTV_PT_.**
4. **R 6cm: a dose auxiliary ring that was made by the external expansion of 6 mm from PTV_LN_ subtracting PTV_LN_.**
5. **R 12cm: a dose auxiliary ring that was made by the external expansion of 12 mm from PTV_LN_ subtracting the external expansion of 6 mm from PTV_LN_.**
